# Iodine-induced thyroid dysfunction: a scientometric study and visualization analysis

**DOI:** 10.3389/fendo.2023.1239038

**Published:** 2023-09-20

**Authors:** Boshen Gong, Xichang Wang, Chuyuan Wang, Wanyu Yang, Zhongyan Shan, Yaxin Lai

**Affiliations:** Department of Endocrinology and Metabolism, Institute of Endocrinology, National Health Council (NHC) Key Laboratory of Diagnosis and Treatment of Thyroid Diseases, The First Affiliated Hospital of China Medical University, Shenyang, Liaoning, China

**Keywords:** iodine, pregnant women, hyperthyroidism, thyroid dysfunction, hypothyroidism, scientometric study, visualization analysis

## Abstract

**Objective:**

Iodine is essential in thyroid hormone production. Iodine deficiency is associated with serious complications (i.e miscarriage and stillbirth), whereas excess can cause thyroid dysfunction (i.e hyperthyroidism, hypothyroidism, thyroid autoimmunity). We conducted this scientometric study to visualize hot spots and trends in iodine-induced thyroid dysfunction over past two decades. The aim of this paper was to help scholars quickly understand the development and potential trend in this field, and guide future research directions.

**Methods:**

Articles on iodine-induced thyroid dysfunction from 2000 to 2022 were retrieved from the Web of Science Core Collection (WoSCC) using the following search terms: (((((TS=(hypothyroid*)) OR TS=(hyperthyroid*)) OR TS= (“TSH deficiency”)) OR TS= (“thyroid stimulating hormone deficiency”)) AND TS=(Iodine)) NOT TS=(radioiodine). Only publications in English were selected. CiteSpace, VOSviewer, Tableau, Carrot2, and R software were used to analyze the contribution and co-occurrence relationships of different countries, institutes, keywords, references, and journals.

**Results:**

A total of 2986 publications from 115 countries and 3412 research institutions were included. From 2000 to 2022, research on iodine-induced thyroid dysfunction progressed over a three-stage development period: initial development (2000-2009), stable development (2010-2016), and rapid development (2016-2022) period. The *Journal of Clinical Endocrinology and Metabolism* had the most co-citations followed and China Medical University (n=76) had the most publications. The top three clusters of co-citation references were isolated maternal hypothyroxinemia, subclinical hyperthyroidism, and brain development. Various scientific methods were applied to reveal acknowledge structure, development trend and research hotspots in iodine-induced thyroid dysfunction.

**Conclusion:**

Our scientometric analysis shows that investigations related to pregnant women, epidemiology surveys, and iodine deficiency are promising topics for future iodine-induced thyroid dysfunction research and highlights the important role of iodine on thyroid function.

## 1 Introduction

Adequate iodine intake is necessary for normal thyroid function (1, 2). Both iodine deficiency and excess lead to thyroid dysfunction, such as hypothyroidism and hyperthyroidism, which is a result of supraphysiologic iodine exposure. The primary source of iodine is diet via the consumption of foods containing iodine, including salt, fruit, and seafood. The recommended daily iodine intake is 150 μg in adults who are not pregnant (3). Iodine status is a key determinant of thyroid dysfunction in both newborns and adults, especially pregnant women, because thyroid hormones are essential for central nervous system development (4, 5). In mild iodine deficiency, the thyroid can compensate for low iodine intake and maintain euthyroidism, while severe iodine deficiency causes hypothyroidism when iodine concentrations are too low for the thyroid gland to produce sufficient thyroid hormone for the body (6, 7). The risk of overt hypothyroidism in one’s life is approximately 5%, and this disease is usually preceded by subclinical hypothyroidism (8). The most common symptoms of hypothyroidism are fatigue, lethargy, cold intolerance, and weight gain (9, 10). In recent years, many studies and guidelines have focused on the management of iodine intake in pregnant women, as they remain at risk of iodine deficiency owing to increased iodine requirements during gestation (11, 12).

Iodine deficiency remains an ongoing problem. Although the recent implementation of salt iodization has significantly reduced the effects of iodine deficiency worldwide, geographical differences and environmental factors still result in various incidences and prevalence thyroid dysfunction in different countries or regions (13, 14). Since mandatory universal salt iodization (USI) was implemented in China 20 years ago, the Chinese population has been consecutively exposed to iodine nutrition status of excessive iodine intake for 5 years (1996– 2001), more than adequate iodine intake for 10 years (2002–2011), and adequate iodine intake for 5 years (2012–2016) (15). A cohort study including three regions with different levels of iodine intake in China showed that more than adequate or excessive iodine intake may lead to hypothyroidism and autoimmune thyroiditis (16).

Research related to iodine-induced thyroid dysfunction has emerged rapidly in recent years (17). An accurate understanding of the research progress and academic trends is of great importance. However, no studies have systematically analyzed the related literature. Scientometric analysis has the advantage of quickly identifying critical issues in a field of interest and guiding future research (18, 19). CiteSpace and VOSviewer are bibliometric analysis software programs that use mathematical and statistical methods to generate and analyze networks of co-cited references based on bibliographic records retrieved from the Web of Science (WOS) (20–22). This study explores the hotspots and development trends of iodine-induced thyroid dysfunction over the past decade using scientometric methodologies and visualization tools.

## 2 Materials and methods

### 2.1 Data collection

In this study, relevant literature was retrieved from the Web of Science Core Collection database (WoSCC) using the following search strategy: ((((((TS=(hypothyroid*)) OR TS=(hyperthyroid*)) OR TS= (“TSH deficiency”)) OR TS= (“thyroid stimulating hormone deficiency”)) AND TS=(iodine)) NOT TS=(radioiodine). The WOS citation database is an information retrieval platform developed by Thomson Reuters in the US (23). This search strategy was limited to published English papers and the literature types were “article” and “review.” Publications from 2000 to 2022 were selected. We only investigated iodine status from environment factors, thus we excluded radioiodine therapy such as iodine-131. Two authors independently conducted a literature search that strictly adhered to the inclusion and exclusion criteria. All searches were completed and downloaded in 1 day to avoid bias caused by daily database updates. Initially, a total of 3381 documents were retrieved, and 393 irrelevant articles, including meeting abstracts, letters, retractions, proceedings papers, and non-English papers, were excluded. A sample of 2988 publications were explored, saved as plain text files, stored in the form of download_txt, and imported to CiteSpace 6.1. R3(Chaomei Chen, Drexel University). After removing duplicates via CiteSpace, 2986 were subjected to visualization analysis (**Figure S1**).

### 2.2 Bibliometric analysis and visualization

CiteSpace and VOSviewer software are powerful complementary science mapping analysis tools (24). The Citespace software is a Java-based application created by Professor Chen of Drexel University in 2004 (25). It can be used to predict research trends and focus on keyword analysis, cluster analysis, author information, and co-citation analysis (26). In this study, we used the following Citespace parameters: time slicing (2000–2022) and year per slice. We selected different node types based on the type of analysis. VOSviewer is a freely available program for constructing and viewing bibliometric maps (27). VOSviewer (Leiden University, Netherlands, version 1.6.18) was used to visualize the collaborations between countries, institutions, and journals. Microsoft Office Excel 2019 was used to analyze the trend of articles published per year and Tableau desktop personal (version 2022.3.1, Tableau Software, Inc.) to show the publication output among different countries by creating interactive visualizations. Carrot2(San Diego workbench-3.10.3) is software that can be used to organize retrieved articles into topics based on the title. Carrot2 was used to analyze recent theme clusters from the retrieved articles. The Bibliometricx package (2.0) in R was used to build a thematic map of the study on iodine-induced thyroid dysfunction, which was divided into four quadrants (Q1 to Q4). The R package was developed by Aria and Cuccurullo and written in R (28).

## 3 Results

### 3.1 Trends analysis of publication output

**Figure 1:**
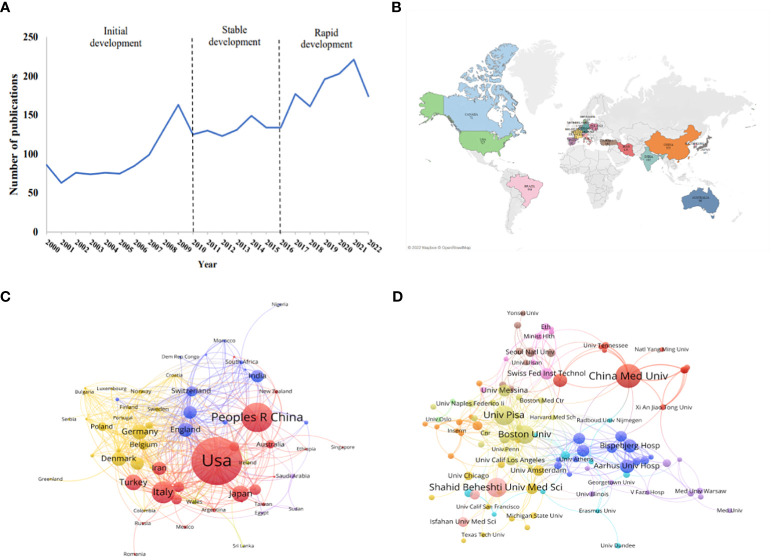
Publication trends on iodine-induced thyroid dysfunction and distribution of countries/regions and Institutions. **(A)** Number of articles published on iodine-induced thyroid dysfunction from 2000 to 2022. **(B)** World map of the top 20 countries devoted to the research on this topic. **(C)** Country cooperation network visualization map. **(D)** The visualization map of collaborations between different institutions.

As shown in **Figure 1A**, the annual distribution of published articles reflects the developmental trend of research related to iodine-induced thyroid dysfunction. The historical development of related research can be divided into three stages: initial development (2000-2009), stable development (2010-2016), and rapid development (2016-2022). In the initial stage, the number of articles steadily increased, demonstrating that people started to focus on this field. At the stable development stage, the publication trend was quite smooth and was maintained at approximately 130 publication outputs per year. Studies on the management of iodine supplementation during pregnancy and postpartum emerged during this stage. In the rapid development stage, scholars showed great interest in this field, the number of published articles continued to increase, with publication outputs reaching 221 in 2021.

### 3.2 Distribution of countries/regions and institutions analysis

A total of 2986 articles were published in 115 different countries. The map in **Figure 1B** shows the top 20 countries with publications on iodine-induced thyroid dysfunction. The top 3 countries/regions were the US (n=646, 21.63%), China (n=336, 11.25%), and Italy (n=225, 7.54%) (**Figure S2**). These top three countries contributed 40.42% of the total number of publications, which was significantly higher than that of other countries. The VOSviewer software was used to visualize the network of countries with more than five published articles, and 66 countries were identified as having met this set threshold. As seen in **Figure 1C**, each node represents a country or region, and the size of the node is proportional to the number of articles published. The lines between the nodes represent the cooperation between countries. A similar color indicates that they are in the same cluster, and there are four clusters in this field. The USA was the main country that focused on iodine-induced thyroid dysfunction, with a total strength of 301, indicating that it cooperated more closely with other countries. Among all lines, those connecting the USA and China were the thickest, indicating that they are closely connected.

From 2000 to 2022, 3412 research institutions were involved in iodine-induced thyroid dysfunction. **Figure 1D** shows a cooperation network map of the research institutions. The threshold was set to more than 9 publications, and 108 institutions reached this threshold. The color of the node represents the cluster to which the institution belongs. There were 108 nodes, 11 clusters, and 360 links. We listed the top 10 institutions/affiliations with the most documents, accounting for 15.30% of the total publications (**Table 1**). China Medical University had the highest number of published articles (n=80), followed by University Pisa (n=61), and Shahid Beheshti University of Medical Sciences (n=60). Among the 3412 institutions, 3315 institutions published fewer than 10 papers, indicating that a few institutions lead the research direction. Although Shahid Beheshti University of Medical Sciences is one of the top 3 institutions with the most articles, its Citation/Publication is 14.27, which is lower than that of other institutions. The top 3 institutions are the Swiss Federal Institute of Technology, Aalborg University Hospital, and Aarhus University Hospital, with values of 89.63, 82.16, and 74.08, respectively, based on the value of citation/publication.

**Table 1:** The top 10 major research institutions related to iodine-induced thyroid dysfunction.

Rank	Organization	Country	Publication	Percentage	Citation	Citation/ Publication
**1**	China Med Univ	China	80	2.68%	2685	33.56
**2**	Univ Pisa	Italy	61	2.04%	2667	43.72
**3**	Shahid Beheshti Univ Med Sci	Iran	60	2.01%	856	14.27
**4**	Boston Univ	USA	55	1.84%	3594	65.35
**5**	Aarhus Univ hosp	Denmark	37	1.24%	2741	74.08
**6**	Bispebjerg Hosp	Copenhagen	36	1.21%	2216	61.56
**7**	Tianjin Med Univ	China	36	1.21%	640	17.78
**8**	Aalborg Univ Hosp	Denmark	31	1.04%	2547	82.16
**9**	Univ Messina	Italy	31	1.04%	1088	35.10
**10**	Swiss Fed Inst Technol	Swiss	30	1.00%	2689	89.63

### 3.3 Analysis of authors co-operation network

**Figure 2:**
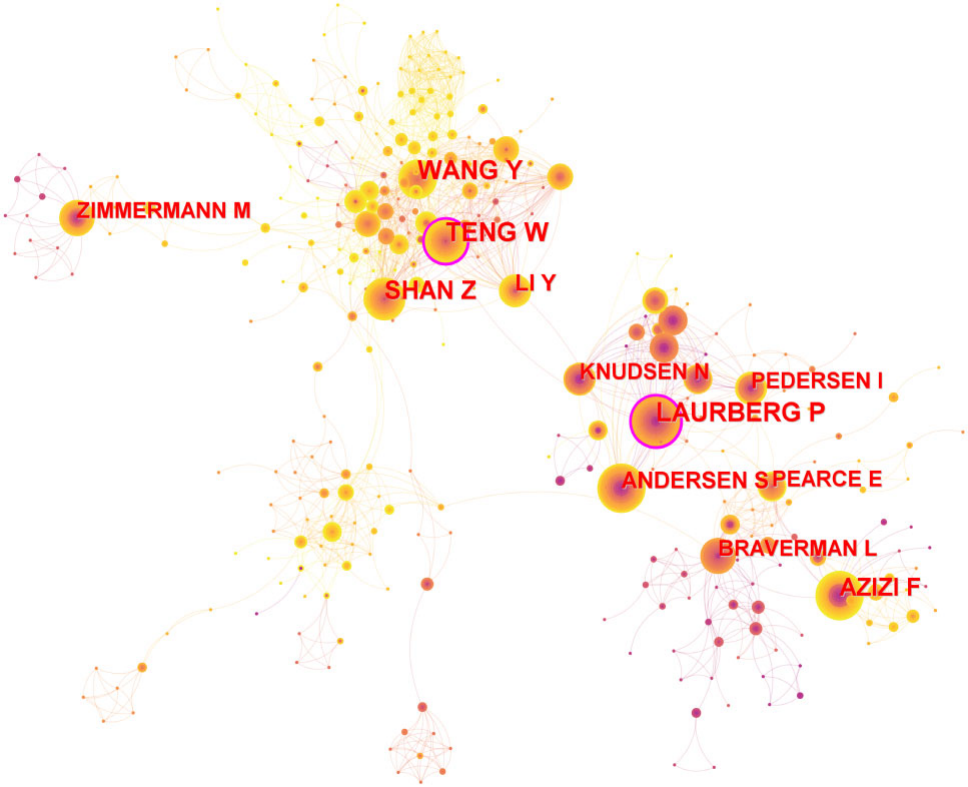
Authors co-operation network. Node size represents the number of publications and the lines between nodes represent cooperation between authors.

A total of 833 authors were involved in this network, with 1996 links and a density of 0.0058 (**Figure 2**). Each node represents an author and the size of the node represents the number of published articles. The two nodes with purple circles indicate that Teng W and Laurberg P have strong centrality, which represents the author’s great influence in this field. The lines in the network represent the authors’ partnerships. There are two main clusters with these two authors in the center of the cluster. As shown in **Table 2**, the top 3 authors with the highest number of published articles were Wang Y (n=64), Laurberg P (n=59), and Teng W (n=57). Based on centrality, the top authors were Teng W (0.11) and Laurberg P (0.11). The number of neighboring nodes represents the cooperation strength. Three Chinese authors, Teng W, Shan Z, and Wang Y, had the most neighboring nodes, 37, 36, and 33, respectively.

**Table 2:** The top 10 authors contributing to publications on iodine-induced thyroid dysfunction.

Rank	Author	Count	Centrality	Neighbouring Nodes	Year
**1**	LAURBERG P	67	0.11	28	2000
**2**	TENG W	64	0.11	37	2005
**3**	WANG Y	61	0.03	33	2009
**4**	SHAN Z	55	0.01	36	2005
**5**	AZIZI F	48	0.03	21	2000
**6**	LI Y	38	0.01	32	2005
**7**	ANDERSEN S	37	0.02	17	2000
**8**	KUNDSEN N	33	0.01	16	2000
**9**	PEDERSEN I	33	0.00	12	2001
**10**	ZIMMERMANN M	32	0.02	19	2002

### 3.4 Hotspots and frontiers analysis

**Figure 3:**
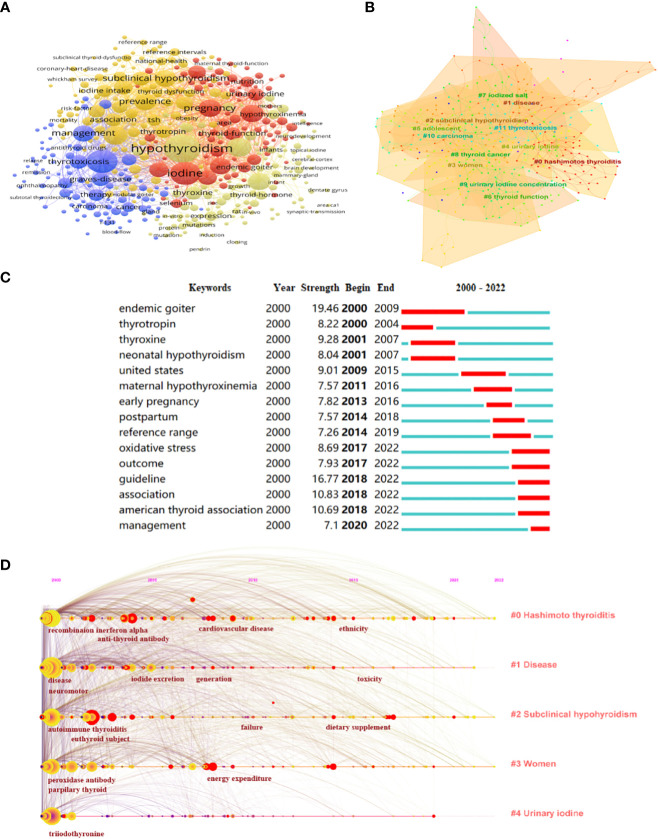
Hotspots and Frontiers Analysis on iodine-induced thyroid dysfunction. **(A)** A network map of keywords. **(B)** Clustering analysis of keywords. **(C)** Top 15 keywords with the strongest citation bursts related to iodine-induced thyroid dysfunction during the past two decades. **(D)** The timeline view of top 5 keywords related to iodine-induced thyroid dysfunction research from 2000 to 2022.

Keyword cooccurrence analysis is common in scientometric analysis (29). Through the analysis of keywords, hotspots in the field can be quickly identified. VOSviewer was used to construct a network map of keywords (**Figure 3A**). The threshold was set to occurrences of more than 11 times, and 399 items reached the threshold. The larger the node, the higher the frequency, and the lines between nodes represent the cooccurrence of keywords. Research on iodine deficiency is mainly associated with hypothyroidism and goiter, particularly in pregnant women and children. To refine the research topics more intuitively and effectively in this field, CiteSpace was used to show the top 12 cooccurrence clusters of keywords (**Figure 3B**). The emergence and bursting of keywords were also analyzed (**Figure 3C**). Endemic goiter was an emergent word from 2000 to 2009, while in recent years, the American Thyroid Association (ATA) and its related guidelines on iodine-induced thyroid dysfunction have been an emergent topic (2018–2022). The timeline viewer is based on the interaction and mutation relationship between keywords, which is helpful for exploring the evolution track (30) (**Figure 3D**). Keywords included “hashimoto thyroiditis”, “disease”, “subclinical hypothyroidism”, “women”, and “urinary iodine” have been top five hotspots since 2000 to 2022 in this field.

### 3.5 Co-cited references and references burst

**Figure 4:**
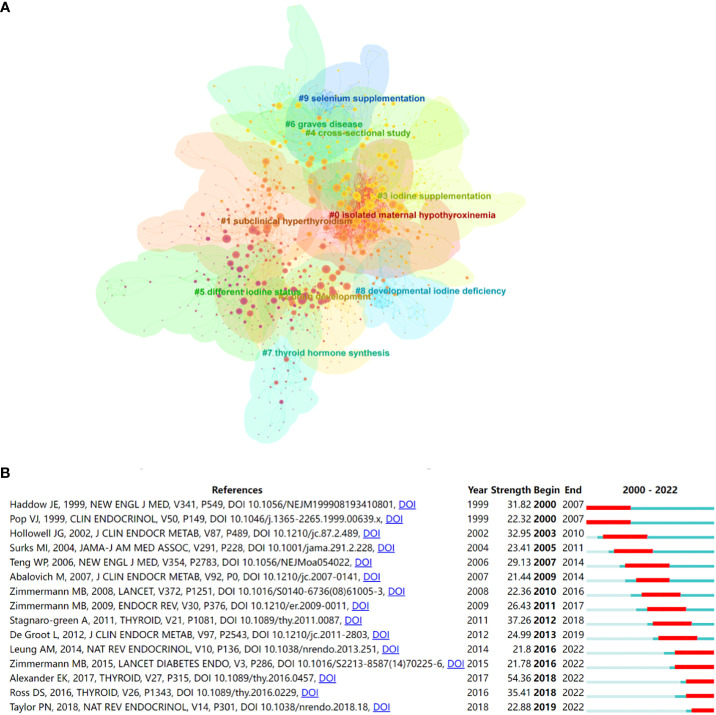
**(A)** The co-citation clusters of references related to iodine-induced thyroid dysfunction. **(B)** Top 15 references with the strongest citation bursts related to iodine-induced thyroid dysfunction from 2000 to 2022.

When two publications are jointly cited by a third publication, this is referred to as a co-citation relationship. Co-citation references are typically regarded as a knowledge base in a particular field (31). A total of 1246 co-cited references were identified from 2000 to 2022 based on CiteSpace and these showed significant modularity and silhouette scores (Q= 0.7405; S= 0.9078), with the time slice was set as a year (pruning: none). The top 10 clusters in this network of co-citation references are shown in **Figure 4A**. The top 3 clusters of co-citation references were isolated maternal hypothyroxinemia, subclinical hyperthyroidism, and brain development. The top 10 co-cited references from 2000 to 2022, ranked by count, are listed in **Table 3**. The top 3 cited references were the practice guidelines published by ATA on the management of iodine nutrition for during pregnancy and the postpartum period (2, 32, 33). Undoubtedly, these publications have been extremely significant in research on iodine-induced thyroid dysfunction. Research trends can be obtained in certain fields by performing burst detection analysis. **Figure 4B** shows the top 15 references with the strongest citation bursts. Red segments represent “burst” year, and the blue segment represents the time interval.

**Table 3:** The top 10 co-cited references in the research of iodine-induced thyroid dysfunction.

Publication Year	First Author	Title	Type	Journal	Citation Counts	Centrality	2022 Impact factor
2017	Alexander EK	2017 Guidelines of the American Thyroid Association for the Diagnosis and Management of Thyroid Disease During Pregnancy and the Postpartum	Review	*Thyroid*	148	0.01	6.506
2011	Stagnaro-green A	Guidelines of the American Thyroid Association for the diagnosis and management of thyroid disease during pregnancy and postpartum	Review	*Thyroid*	132	0.02	6.506
2016	Ross DS	2016 American Thyroid Association Guidelines for Diagnosis and Management of Hyperthyroidism and Other Causes of Thyrotoxicosis	Review	*Thyroid*	103	0.03	6.506
2012	De Groot L	Management of thyroid dysfunction during pregnancy and postpartum: an Endocrine Society clinical practice guideline.	Review	*J Clin Endocr Metab*	95	0.01	6.134
2006	Teng WP	Effect of iodine intake on thyroid diseases in China	Article	*New England J Med*	90	0.04	176.079
2014	Leung AM	Consequences of excess iodine	Review	*Nat Rev Endocrinol*	89	0.00	47.564
2013	Bath SC	Effect of inadequate iodine status in UK pregnant women on cognitive outcomes in their children: results from the Avon Longitudinal Study of Parents and Children (ALSPAC)	Article	*Lancet*	88	0.02	202.731
2011	Bahn RS	Hyperthyroidism and other causes of thyrotoxicosis: management guidelines of the American Thyroid Association and American Association of Clinical Endocrinologists.	Review	*Thyroid*	80	0.04	6.506
2015	Zimmermann MB	Iodine deficiency and thyroid disorders	Review	*Lancet Diabetes Endo*	78	0.02	44.867
2009	Hollowell, JG	Serum TSH, T(4), and thyroid antibodies in the United States population (1988 to 1994): National Health and Nutrition Examination Survey (NHANES III)	Review	*J Clin Endocr Metab*	77	0.05	6.134

### 3.6 Journals and co-cited academic journals analysis

**Figure 5:**
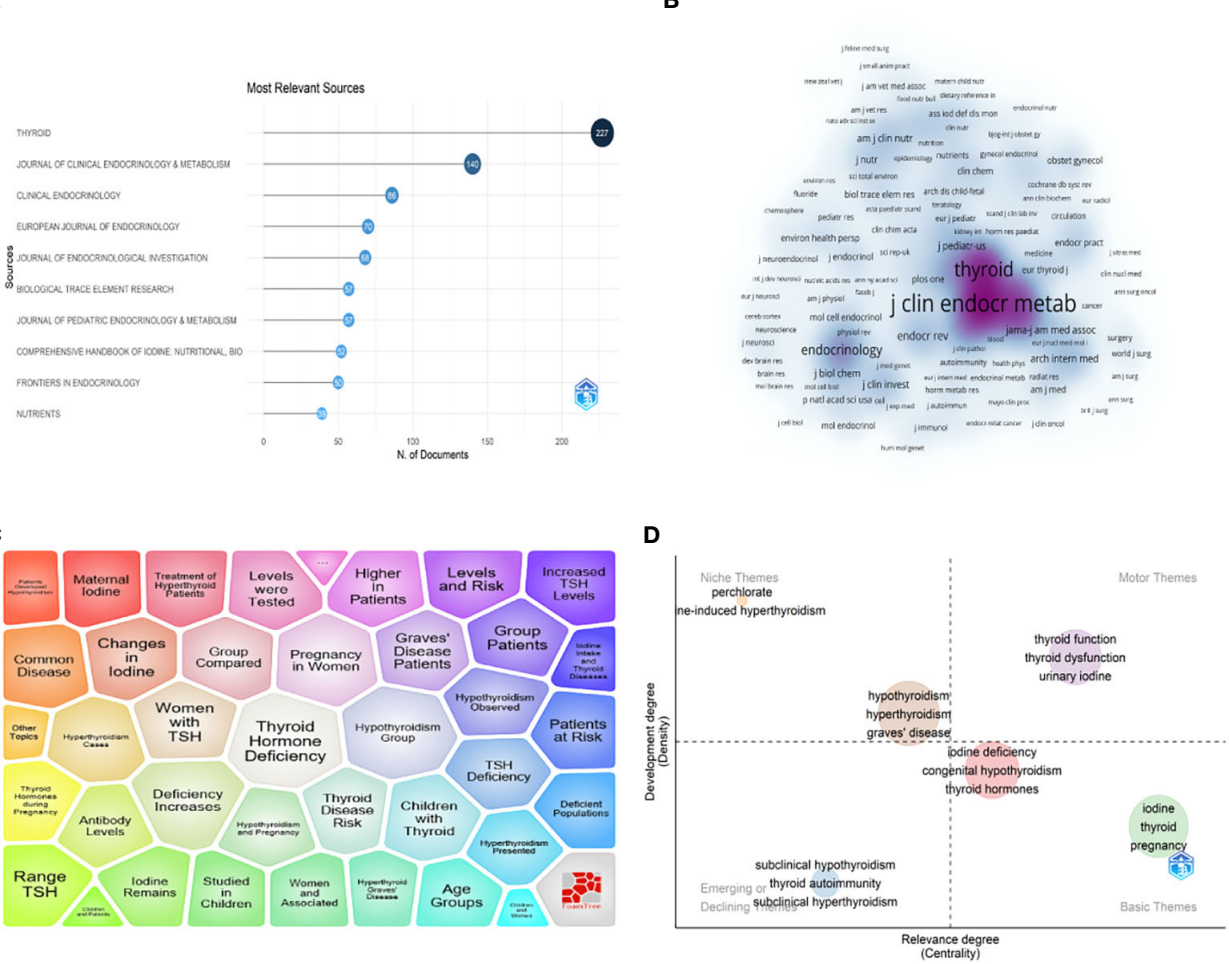
**(A)** Top 10 journals related to iodine-induced thyroid dysfunction based on publication number. **(B)** Journal density map based on Co-citation analysis results by VOSviewer. **(C)** Major topic analysis on iodine-induced thyroid dysfunction based on the Carrot2 software. **(D)** Thematic map of the field of iodine-induced thyroid dysfunction.

A total of 2986 articles on iodine-induced thyroid dysfunction were published in 952 different journals from 2000 to 2022. As shown in **Figure 5A**, *Thyroid* had the highest number of output and impact factors (227, 7.60%, IF=6.506), followed by *Journal of Clinical Endocrinology and Metabolism* (140, 4.69%, IF=6.134) (**Table 4**). The top 10 journals contributed 28.33% of the total publication output in this field. To define the influence journals in this field, the VOSviewer software was also used to show a density map based on journal contributions. The minimum number of citations of a source was set to more than 46, and 368 journals met the threshold (**Figure 5B**). In an item density map, the density of an element depends on the number and weight of its surrounding elements. Specifically, the journal with the highest number of citations was the *Journal of Clinical Endocrinology and Metabolism* (n=12751), followed by *Thyroid* (n=8645).

**Table 4:** The top 10 journals ranked by number of publications.

Journal	Documents	Percent	Citations	2022 impact factor	2022 JCR partition	H-index
*Thyroid*	227	7.60%	8645	6.506	Q1	142
*Journal of Clinical Endocrinology and Metabolism*	140	4.69%	12751	6.134	Q1	353
*Clinical Endocrinology*	86	2.88%	4437	3.523	Q3	147
*European Journal of Endocrinology*	70	2.34	3581	6.558	Q1	148
*Journal of Endocrinological Investigation*	68	2.28%	1716	5.467	Q2	84
*Biological Trace Element Research*	57	1.91%	466	4.081	Q3	80
*Journal of Pediatric Endocrinology and Metabolism*	57	1.91%	518	1.52	Q4	65
*Comprehensive Handbook of Iodine: Nutritional, Biochemical*	52	1.74%	54	_	_	_
*Frontiers in Endocrinology*	50	1.67%	276	6.055	Q1	68
*Nutrients*	39	1.31%	443	6.706	Q1	115

### 3.7 Major topics and thematic analysis

Carrot2 software was used to analyze the major topic in the field of iodine-induced thyroid dysfunction (**Figure 5C**). The top 4 topic words were “Thyroid Hormone Deficiency” (416 documents), “Hypothyroidism Group” (402 documents), “Deficiency Increases” (385 documents), and “Pregnancy in Women” (366 documents). The Bibliometricx package (2.0) in R was used to build a thematic map of studies on iodine-induced thyroid dysfunction. As shown in **Figure 5D**, the horizontal axis represents cluster centrality and the vertical axis represents cluster density. Thus, we can observe that: (a) in the upper right quadrant, there are well-developed and essential topics in the research area; (b) in the upper left quadrant, highly developed themes and isolated themes; (c) emerging or declining themes appear in the lower left quadrant; and (d) in the lower right quadrant, there are general, transversal themes. Importantly, studies on iodine-induced thyroid dysfunction related to iodine deficiency and pregnancy are promising.

## 4 Discussion

To better understand the development, knowledge structure, and development trend of iodine-induced thyroid dysfunction, a scientometric analysis using Citespace, VOSviewer, Tableau, Carrot2, and Bibliometricx package in R was performed (34, 35). Research on iodine-induced thyroid dysfunction has been through a three-stage development period from 2000 to 2022 (initial development [2000–2009], stable development [2010-2016], and rapid development [2016-2022]). Based on the results of keyword burst citation analysis, it was revealed that the research in this field was mainly focused on the iodine status of infants in the initial development stage (36, 37), while in the stable development stage publication on the management of iodine supplementation during pregnancy and the postpartum period predominated (38). In recent years, ATA has shown great interest in iodine-induced thyroid dysfunction (39, 40), and has revised several practice guidelines, which significantly influenced on the management of iodine intake, especially during pregnancy (41–43). From the distribution seen in country analysis, it can be seen that China contributed 353 publications to this field, accounting for 10.71% of the total publications. This might be because China suffers from mild to moderate iodine deficiency, and China implemented universal salt iodization legislation nationally in 1996 (15).

The research on iodine-induced thyroid dysfunction located in Q1 of the thematic analysis had high centrality and density, indicating that this field had a relatively mature level of research. Thyroidal iodine uptake is regulated by the sodium-iodine symporter (NIS) and thyroglobulin (44, 45). Thyroid hormone biosynthesis requires iodide uptake into the thyrocytes, and efflux into the follicular lumen through NIS, where it is combined with selected tyrosyls of thyroglobulin. Iodine-induced hyperthyroidism in the early 20th century was a consequence of iodine supplementation through dietary salt (46). Thyroid dysfunction due to excess iodine intake is usually mild and transient; however, iodine-induced hyperthyroidism can be life-threatening (47–49). Excess iodine may lead to excessive thyroid hormone synthesis and release, inducing the development of iodine-induced hyperthyroidism, especially in iodine-rich areas (50). In iodine-sufficient areas, euthyroid patients previously treated for Graves’ disease are prone to developing iodine-induced hyperthyroidism with excess iodine intake (51). A cohort study conducted in three regions in China with different levels of iodine intake showed that more than adequate or excessive iodine intake may lead to hypothyroidism and autoimmune thyroiditis (16). Autoimmune thyroiditis, specifically, can result from excess iodine intake possibly because of inflammasome activation (52, 53). This is supported by an animal study, where NOD.H2h4 mice developed autoimmune thyroiditis after being fed 0.05% sodium iodide in drinking water for 8 and 16 weeks (54).

Iodine-induced thyroid dysfunction can be overt or subclinical (48). Subclinical hypothyroidism affects 4–20% of the population living in iodine-sufficient areas (55). Subtle maternal thyroid hormone deficiency in pregnant women may negatively impact newborn growth, and maternal subclinical hypothyroidism is the main cause of poor neurodevelopment in the offspring (56). A cross-sectional study conducted in China showed that iodine supplementation after birth does not reverse the neurological damage that results from maternal or subclinical hypothyroidism (57). Besides, there still exists a challenge in diagnosing subclinical hypothyroidism in pregnant women (58). A study conducted in Korea found that excessive iodine intake from breast milk may lead to subclinical hypothyroidism in infants (59). Thus, appropriate measurements should be performed to monitor pregnant women with excessive iodine intake (60). A comparative cross-sectional and longitudinal survey in China showed that subclinical hyperthyroidism is more prevalent in iodine-deficient areas than in iodine-excessive area (61). A study exploring the association between thyroid and iodine status in children and adolescents with chronic autoimmune thyroiditis showed that 36.6% of patients had subclinical hypothyroidism and 8.5% had subclinical hyperthyroidism (62).

Based on the results of the thematic analysis, it can be observed that research related to pregnant women’s iodine status may be promising and needs to be further developed and improved. Adequate maternal dietary intake of iodine during pregnancy is essential for maternal thyroxine production, and later for thyroid function and central nervous system development in newborns (63–65). The ATA revised the practice guidelines for the management of thyroid disease during pregnancy and the postpartum period (2). The ideal dietary allowance of iodine recommended by the World Health Organization is 200 μg per day for pregnant women (66). Because iodine is an essential nutritional supplement for breastfed infants, lactating women should increase dietary iodine requirements (66, 67). A perspective cohort study conducted in China showed maternal iodine insufficiency and excess during early pregnancy could both adversely affected fetal growth including maternal and fetal goiter, cretinism, intellectual impairments (68, 69). The implementation of salt iodization has significantly reduced iodine deficiency, but during pregnancy, most women still need an iodine-containing supplement (70). Most women in Europe are iodine deficient during pregnancy, while less than 50% receive supplementation with iodine Besides, mild iodine deficiency was also observed in the pregnant women in Finland, and Saudi Arabia (71, 72).

At the population level, excess iodine intake may arise from the consumption of over-iodized salt, drinking water, iodine-containing dietary supplements, excess seaweed, or a combination of these sources (73, 74). More than two billion people worldwide suffer from thyroid disorders due to either iodine deficiency or excess (75, 76). The median urinary iodine concentration of a population reflects the total iodine intake from all sources and can accurately identify iodine status (77). Thus, a large cross-sectional study is required to explore the iodine status of the population. A USA study conducted from 1988 to 1994 involving 13,344 individuals that explored thyroid functions and nutritional status showed the strongest centrality (78). A national cross-sectional study conducted in China, which included 78470 participants showed that iodine excess was associated with higher odds of overt hyperthyroidism and subclinical hypothyroidism (15, 79). Ma et al. revealed the spatial responses of thyroid dysfunction risks to iodine in continental groundwater by creating a national map of groundwater iodine levels throughout China (80). A multicenter cross-sectional study conducted in East Africa found that chronic excess iodine intake could be well-tolerated by women, infants, and children (81).

### 4.1 Strengths and limitations

Strengths of this study include the use of various scientific methods, including CiteSpace, VOSviewer, and the Bibliometricx package in R. However, this scientometric study had some limitations. First, only the WoSCC database was used, which resulted in some articles that were inevitably missing. Second, iodine may be associated with thyroid disorders with euthyroid function, such as thyroid nodules or thyroid cancer, which were excluded from the study. Third, only publications written in English were included, which may have led to a linguistic bias.

## 5 Conclusions

To the best of the authors’ knowledge, this study is the first comprehensively performed a scientometric analysis exploring the research trends and hotspots of iodine-induced thyroid dysfunction from 2000 to 2022. The development of iodine-induced thyroid dysfunction has progressed through a three-stage development period from 2000 to 2022. Research related to pregnant women, epidemiology surveys, and iodine deficiency showed a promising future in the field of iodine-induced thyroid dysfunction by scientometric analysis. This study can help scholars quickly identify critical issues in the field of interest and guide future research directions.

## Data availability statement

The original contributions presented in the study are included in the article/**Supplementary Material**. Further inquiries can be directed to the corresponding authors.

## Author contributions

Conceptualization, BG and WY; methodology, XW; software, BG, formal analysis, BG and CW; investigation, YL, re-sources, XW, writing—original, BG; writing—review and editing, XW, WY, ZS, visualization, WY; supervision, ZS, YL, funding acquisition, and project administration CW, ZS. All authors contributed to the article and approved the submitted version.

## Supplementary material

Supplementary Figure 1 Flow chart of screening processes and scientometric analyses.

Supplementary Figure 2 Top 10 productive countries ranked by publications.
