# Supplementary figures and images for: Iodine-induced thyroid dysfunction: a scientometric study and visualization analysis
Source: Front Endocrinol (Lausanne). 2023 Sep 20;14:1239038. doi: 10.3389/fendo.2023.1239038 (PMC10548383; doi:10.3389/fendo.2023.1239038)

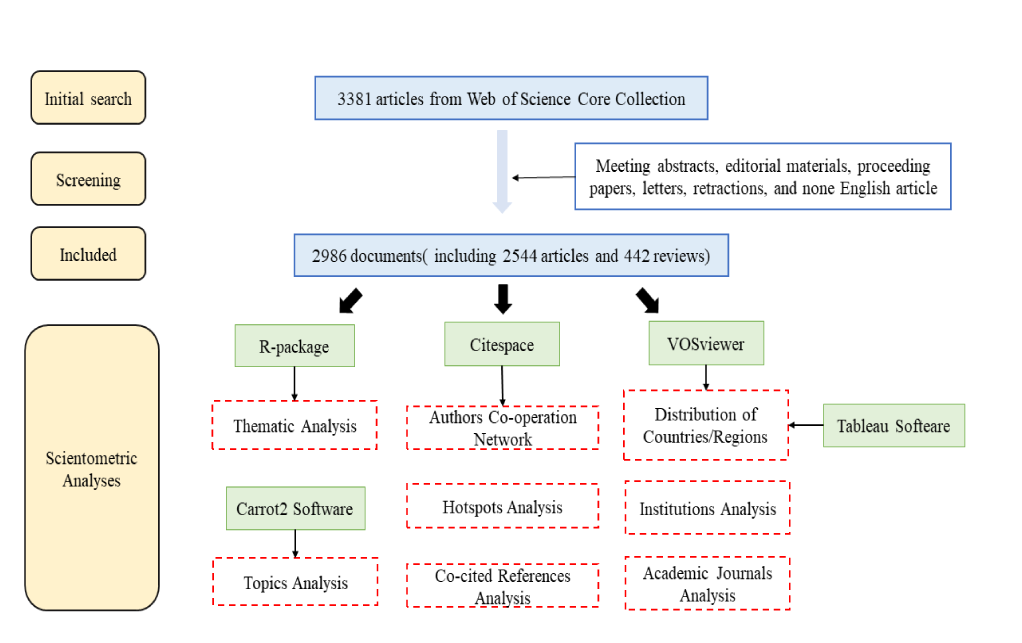

Supplement: Supplementary Figure 1 — Flow chart of screening processes and scientometric analyses. [file Image_1.tif]

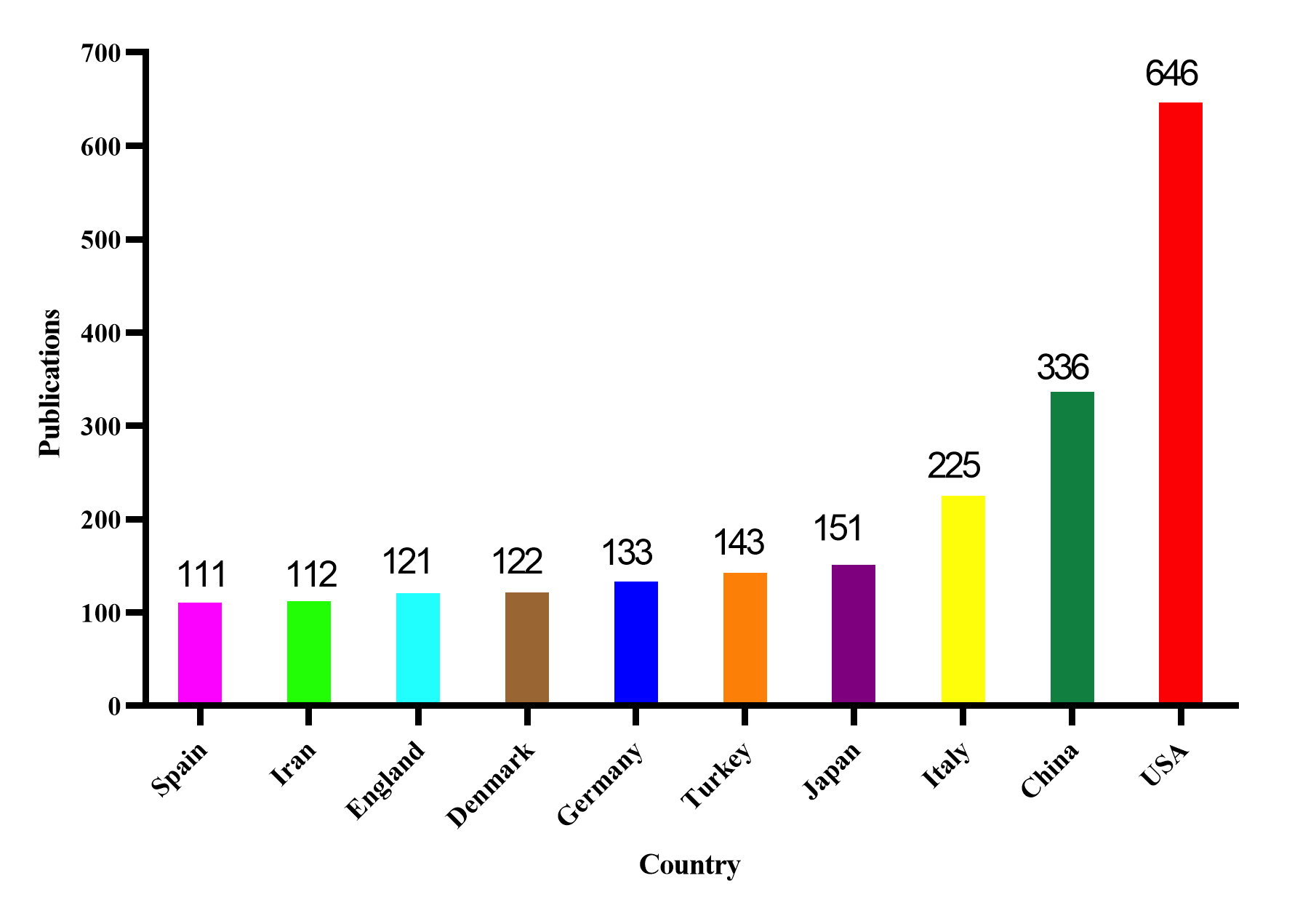

Supplement: Supplementary Figure 2 — Top 10 productive countries ranked by publications. [file Image_2.tif]
